# Identifying MicroRNA-mRNA regulatory network in colorectal cancer by a combination of expression profile and bioinformatics analysis

**DOI:** 10.1186/1752-0509-6-68

**Published:** 2012-06-15

**Authors:** Jihong Fu, Wentao Tang, Peng Du, Guanghui Wang, Wei Chen, Jingming Li, Yunxiang Zhu, Jun Gao, Long Cui

**Affiliations:** 1 Colorectal Surgery Department, Xinhua Hospital, School of Medicine, Shanghai Jiaotong University, Kongjiang Road 1665, Shanghai, 200092, China; 2 Second Affiliated Hospital, Yangzhou University, Jiangsu, 225001, China; 3 Department of Gastroenterology, Changhai Hospital, Second Military Medical University, Changhai Road 168, Shanghai, 200433, China

**Keywords:** MicroRNAs, Target mRNAs, Colorectal cancer, Microarray

## Abstract

****Background**:**

MicroRNAs (miRNAs) are involved in carcinogenesis and tumor progression by regulating post-transcriptional gene expression. However, the miRNA-mRNA regulatory network is far from being fully understood. The objective of this study is to identify the colorectal cancer (CRC) specific miRNAs and their target mRNAs using a multi-step approach.

****Results**:**

A multi-step approach combining microarray miRNA and mRNA expression profile and bioinformatics analysis was adopted to identify the CRC specific miRNA-mRNA regulatory network. First, 32 differentially expressed miRNAs and 2916 mRNAs from CRC samples and their corresponding normal epithelial tissues were identified by miRNA and mRNA microarray, respectively. Secondly, 22 dysregulated miRNAs and their 58 target mRNAs (72 miRNA-mRNA pairs) were identified by a combination of Pearson’s correlation analysis and prediction by databases TargetScan and miRanda. Bioinformatics analysis revealed that these miRNA-mRNAs pairs were involved in Wnt signaling pathway. Additionally, 6 up-regulated miRNAs (mir-21, mir-223, mir-224, mir-29a, mir-29b, and mir-27a) and 4 down-regulated predicted target mRNAs (SFRP1, SFRP2, RNF138, and KLF4) were selected to validate the expression level and their anti-correlationship in an extended cohort of CRC patients by qRT-PCR. Except for mir-27a, the differential expression and their anti-correlationship were proven. Finally, a transfection assay was performed to validate a regulatory relationship between mir-29a and KLF4 at both RNA and protein levels.

****Conclusions**:**

Seventy-two miRNA-mRNA pairs combined by 22 dysregulated miRNAs and their 58 target mRNAs identified by the multi-step approach appear to be involved in CRC tumorigenesis. The results in our study were worthwhile to further investigation via a functional study to fully understand the underlying regulatory mechanisms of miRNA in CRC.

## **Background**

MicroRNAs (miRNAs) are one family of small (~22 nucleotides), non-coding RNAs. They regulate gene expression post-transcriptionally through binding to the complementary sites of target mRNAs in the 3’UTR [1], and play an important role in regulating diverse biological processes, such as stem cell maintenance, development, metabolism, proliferation, differentiation and apoptosis [2]. Cumulative evidence indicates that some miRNAs act as either oncogenes or tumor suppressors in the tumorigenesis of CRC and possess the tumor marker potential for diagnostic, therapeutic, prognostic exploration [3-9]. To elucidate the mechanism of miRNA regulation, it is critical to discover the differentially expressed miRNA and identify the target mRNAs of miRNAs.

Many aberrant expressed miRNAs were found in CRC tissues by miRNA microarray assay [10-15]. The target relationship between particular miRNA and mRNA were identified by in-vitro experiments such as transfection and luciferase activity assays [16-19]. Although in-vitro experiments provide invaluable information on miRNA regulation, those experiments are not possible to be applied to clinical samples, and it is also impossible to obtain results under a genome-wide circumstance. Many databases were explored to predict the targets of miRNAs by either seed sequence complementary or thermodynamic algorithm [20-23], however these databases only provide the possibility of predicting direct target-relationship miRNA-mRNA pairs and can not predict disease-specific pairs in a specific disease.

MiRNAs regulate target gene expression in two ways, mRNA degradation or translation inhibition [24,25]. The target genes which were regulated by miRNAs through mRNA degradation are negatively correlated with the miRNA regulators. Therefore an approach for the identification of miRNA-mRNA regulatory modules was introduced by constructing the paired miRNA-mRNA expression profiles and predicting the putative target genes of miRNAs [26,27]. However, this novel multi-step approach has not been tested in CRC, especially in clinical matched tumor/normal mucosa samples.

In this study, we applied the multi-step approach by constructing the aberrant paired miRNA-mRNA expression profiles and the clusters of miRNA target genes to investigate the role of miRNA in CRC.

## **Results**

### **Strategy**

**Figure 1:**
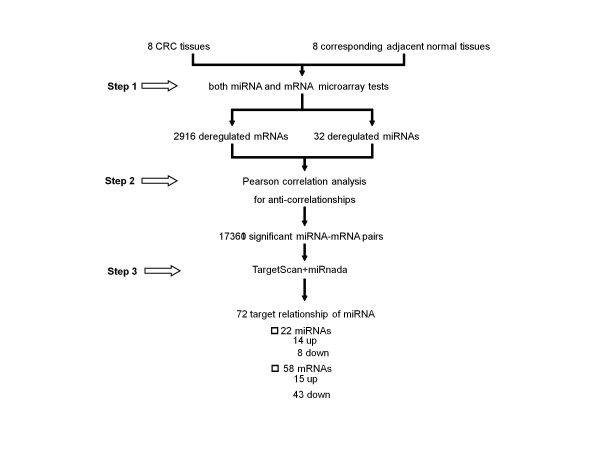
**Step 1, both miRNA and mRNA microarray tests for identifying significantly dysregulated miRNAs and mRNAs in colorectal cancer.** Step 2, Pearson correlation analysis for anti-correlationship of dysregulated miRNAs and mRNAs. Step 3, TargetScan and Mirnada predicting the target-relationship miRNA-mRNA pairs.

As shown in Figure 1, a multi-step approach was adopted to identify the mRNA targets of CRC-specific miRNAs in colorectal cancer. First, in the same cohort of eight cases of pathologically confirmed CRC and their corresponding adjacent normal tissues, the significantly aberrant expression profiles of both miRNA and mRNA were obtained by microarray test. Second, the significant anti-correlation between miRNA-mRNA pairs was determined based on the expression level of each aberrantly expressed miRNA and each aberrantly expressed mRNA. Third, only the target-relationship miRNA-mRNA pairs, which were also predicted by both TargetScanHuman V5.1 [20] and miRanda [23], were retained. Fourth, some of the predicted direct target-relationship miRNA-mRNA pairs were selected to be validated in an extended cohort of CRC tissues by qRT-PCR detection.

Further in detailed explanation, the target genes of dysregulated miRNA in CRC tissues should meet the following requirements: 1)the microRNAs and mRNAs were differentially expressed between CRC tissues and corresponding normal samples 2) the expression level of miRNAs and mRNAs were significantly anti-correlated (P < 0.05) 3) the mRNA is a putative target of miRNA predicted by both TargetScan [20] and miRanda [23].

### **miRNA and mRNA aberrant expression profiles in CRC samples**

**Figure 2:**
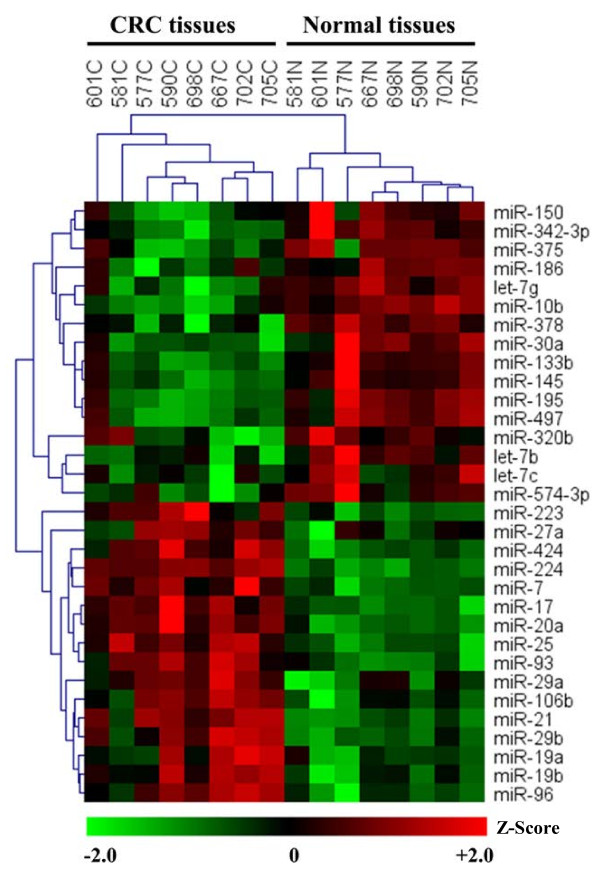
**FDR < 0.05 for students t-test and q-value < 0.05 for SAM were considered as significantly differentially expressed.** The data were z-score transformed and were indicated by the color bar below the heatmap.

The miRNA expression profiles in 8 CRC tissues and their corresponding adjacent normal tissues were determined using miRNA microarray analysis. Histologically, the 8 CRC cases were all moderately differentiated, non-mucinous adeno-carcinoma, micro-satellite stable. The clinical data of the 8 CRC cases are listed in Additional file 1: Table S1. After between-array normalization and filtering on flags (IsGeneDetected, WellAboveNeg) carried out by RMA algorithm [28], 125 miRNAs were selected for further analysis. Using Student’s *t*-test and Significant Analysis of Microarray analyses (SAM) [29] (FDR < 0.05 from student’s t-test and q-value < 0.05 from SAM), 32 miRNAs were found to be significantly differentially expressed between CRC tissues and their corresponding adjacent normal tissues, including 16 up-regulated and 16 down-regulated which were shown in Additional file 1: Table S2. In addition, the aberrantly different miRNA expression levels were illustrated by Heatmap in Figure 2.

The mRNA expression profiles of the same 8 paired-samples in miRNA expression profiling were analyzed by Whole Human Genome Agilent 4 × 44 K Oligonucleotide Microarray (Agilent). After between-array normalization performed by Linear Models for Microarray Data (R-package Limma) [30], 2916 genes were identified as significantly differentially expressed in CRC tissues using the criteria similar to that in miRNA microarray assays (FDR < 0.05 from student’s t-test and q-value < 0.05 from SAM) and was included in Additional file 2: Table S1.

The microarray data was deposited to the NCBI Gene Expression Omnibus (accession GSE35982)

### **Identifying target genes by anti-correlation and target prediction**

Pearson’s correlation analysis was applied to the 32 significantly differentially expressed miRNAs and significantly differentially expressed 2916 mRNAs. Since miRNAs act as negative regulators, up-regulated miRNAs resulted in down-regulated target mRNAs, and vice versa. A total of 17360 miRNA-mRNA pairs (Additional file 3: Table S1) were obtained by two criteria: Pearson’s correlation less than -0.5 was considered as anti-correlation and a p-value less than 0.05 as statistically significant. A multiple-hypothesis correction was carried out by Benjamini-Horchberg algorithm. The FDR value of all the 17630 miRNA-mRNA pairs were less than 0.3 and were selected for further analysis. To predict the target mRNAs for the 32 dysregulated miRNAs, TargetScanHuman (V5.1.) and miRanda database were used. To eliminate false positive rates of the target prediction databases, only the miRNA-mRNA pairs simultaneously predicted by both TargetScanHuman V5.1 and miRanda were considered as target relationship miRNA-mRNA pairs in our study. Seventy-two miRNA-mRNA pairs combined by 22 dysregulated miRNAs and their 58 target mRNAs were predicted by those two prediction databases and simultaneously presented an anti-correlationship between the expression level of miRNAs and mRNAs. These 58 predicted target mRNAs included many genes previously reported to be involved in tumorigenesis of CRC or other malignant tumors such as KLF4 [31], SFRP1 [32], SFRP2 [33], RNF138 [34]. These 72 predicted miRNA-mRNA pairs contained 14 up-regulated miRNAs targeting 43 down-regulated mRNA (Additional file 4) and 8 down-regulated miRNAs targeting 15 up-regulated mRNAs (Additional file 5). The expression levels of 58 target mRNAs were illustrated by Heatmap (Additional file 1: Figure S1).

### **Pathway analysis of the miRNA targets**

To obtain a better understanding of biological function of miRNAs dysregulated in CRC, function analysis of the miRNA target genes was carried out by Database for Annotation, Visualization and Integrated Discovery (DAVID) v6.7 [35]. As a result of finding which biological processes of GO or pathway from the KEGG database were significantly enriched, 38 GO categories were found enriched with a p-value < 0.05 (Additional file 6), however using a cutoff of FDR < 0.05, no GO category were found significantly enriched. Using a cutoff of FDR < 0.05, the Wnt signaling pathway (hsa04310) was the only significantly enriched KEGG pathway (Table 1).

**Table 1:** Wnt signaling pathway was enriched by the miRNA targets

**Wnt signaling pathway was enriched by the targets of miRNAs**						
Term	Fold Enrichment	p- value	FDR (%)	Bonferroni	genes	miRNAs
hsa04310: Wnt signaling pathway	8.418	0.002	1.844	0.0767	WNT5A	hsa-mir -186
					SFRP1	hsa-mir -27a
					SFRP2	hsa-mir -224
					CAMK2D	hsa-mir -7
					CHP2	hsa-mir -224

### **Validation of the expression level of miRNA and mRNA in an extended cohort of CRC tissues by qRT-PCR**

To validate the reliability of the microarray test,6 up-regulated miRNAs (mir-21, mir223, mir224, mir29a, mir29b, and mir27a) and their 4 down-regulated target mRNAs (SFRP1, SFRP2, RNF138, and KLF4) were selected for qRT-PCR examination in 40 matched CRC/normal mucosa tissues whose clinical data were listed in Additional file 1: Table S3. The rationale for selecting these up-regulated miRNAs and down-regulated mRNAs was the down-regulated expression of SFRP1 [32,36], SFRP2 [32,33,37,38], and RNF138 [34] were reported to be involved in the activity of the Wnt receptor signaling pathway. Further it was found that the expression of KLF4 [31] was down-regulated in CRC tissues compared with normal mucosa.

**Figure 3:**
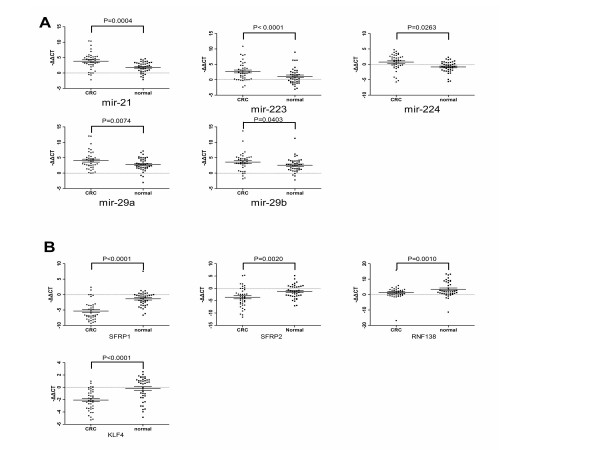
**A. The expression levels of 5 miRNAs were significantly up-regulated. B.** The expression levels of 4 mRNA targets in CRC samples and adjacent normal tissues. The expression levels were expressed as –ΔΔCT after normalized with beta-actin. *P-*value was calculated by paired t-test.

As demonstrated in Figure 3A, the five miRNAs (mir-21, mir-223, mir-224, mir-29A and mir-29B) were significantly up-regulated in CRC tissues, except for mir-27a due to non-efficient amplification (CT value in most of the samples was either higher than 35 or even cannot be defined). As shown in Figure 3B, the expression levels of the 4 mRNAs (SFRP1, SFRP2, RNF138, KLF4)were significantly down-regulated in CRC tissues.

Additionally, the Pearson’s correlation coefficient between the expression levels of miRNA-mRNA pairs were calculated, and two identified miRNA-mRNA pairs, mir-224 and SFRP2, mir-29a and KLF4 were found significantly anti-correlated in qRT-PCR tests. However, 2 miRNA-mRNA pairs (mir-29a and SFRP1; mir-21 and SFRP1) not predicted by the two exist between miRNAs and SFRP1 (Table 2).

**Table 2:** Validation of the anti-correlationship between miRNAs and mRNAsexpression levels by qRT-PCR

	**Correlation (Pearson’s R, *p* value)**			
	**SFRP1**	**SFRP2**	**RNF138**	**KLF4**
mir-21	**Sig.** (−0.24,0.02)	NS-	NS-	NS-
mir-224	NS-	**Sig.**^**a**^ (− 0.64,0.00)	NS-	NS-
mir-29a	**Sig**^**a**^ (− 0.26,0.02)	NS-	NS^**a**^-	**Sig.**^**a**^ (− 0.38,0.00)
mir-29b	NS-	NS-	NS-	NS-

a, predicted target-relationship miRNA-mRNA pairs by TargetScan and miRanda.

Sig., statistic significance, *p* < 0.05.

NS, non statistic significance.

#### **In vitro analysis revealed expression of KLF4 is regulated by mir-29a**

**Figure 4:**
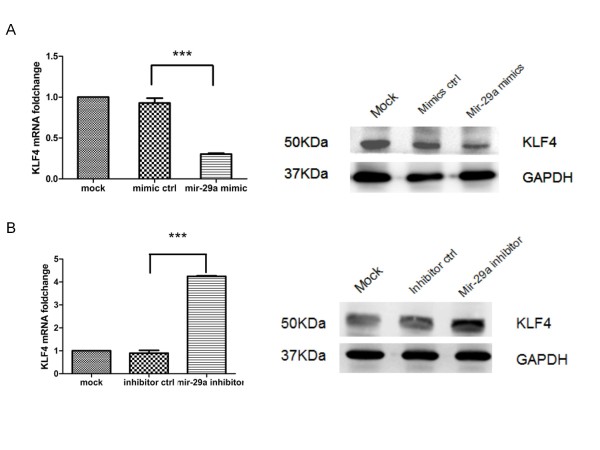
**A. The transfection of mir-29a mimics into HCT-116 cells down-regulates KLF4 mRNA expression (Left, QRT-PCR) and protein expression (Right, Western blot). B.** The transfection of mir-29a inhibitor into HCT-116 cells up-regulates KLF4 mRNA expression (Left, QRT-PCR) and protein expression (Right, Western blot). The experiments were independently repeated three times and GADPH served as control. ***, P < 0.001.

To further validate identified miRNA-mRNA interactions, the transfection assay was carried out to demonstrate the relationship between mir-29a and its identified target, KLF4. HCT-116 cells were transfected with mir-29a mimics and mir-29a inhibitor. The qRT-PCR and Western-blot revealed that the KLF4 mRNA and protein expression were significantly down-regulated by the mir-29a mimics (Figure 4a) while the KLF4 expression levels were significantly up-regulated by mir-29a inhibitor (Figure 4b), compared with the control group and mock group. These data indicated that the KLF4 mRNA and protein expression levels are controlled by mir-29a, suggesting that KLF4 probably was the target of mir-29a.

## **Discussion**

miRNAs, acting as negative regulators of gene expression, regulate various biological processes via inhibiting the expression of their target genes. Increasing evidence shows that some miRNAs play critical roles in tumorigenesis of CRC and have a potential clinical value in diagnosis, treatment and prognosis evaluation for CRC [4-9]. It is of great importance to identify the targets of miRNAs and elucidate their complex regulatory networks.

In this study, a multi-step approach to identify the mRNA targets of dysregulated miRNAs in CRC was adopted. Firstly, in the same cohort of samples containing CRC tissues and their corresponding adjacent tissues, the expression profiles of miRNAs and mRNAs were screened. Secondly, all possible pairs of miRNA-mRNA with an anti-correlationship were identified by Pearson’s correlation analysis, implying that these pairs of miRNA-mRNA may contain both the direct target-relationship and indirect anti-correlationship pairs. Thirdly, the reliability of the identified target-relationship pairs of miRNAs-mRNAs was assessed and confirmed by using both TargetScan and miRanda databases. Finally, the differential expression and their anti-correlationship of 6 miRNAs and 4 mRNAs were validated by qRT-PCR and the regulatory effect of mir-29a on the expression of target of KLF4 mRNA was validated by in-vitro analysis. Through the multi-step approach, 72 miRNA-mRNA pairs containing 22 dysregulated miRNAs and their 58 target mRNAs in CRC were identified, and the Wnt signaling pathway was the only affected pathway based on pathway analysis for the miRNA targets.

Compared with previous studies on the miRNA expression profile of CRC, including miRNA microarray [12-15], miRNA cloning and SAGE [39], most dysregualted miRNAs detected in this study were consistent with at least one of the previous studies. However, some discrepancy and inconsistency of the miRNA expression profile between studies were found. For instance, the elevated expression of mir-29b in CRC tissues in this study and the recent report [14] contradicted findings in Cummins’ report [39] and the elevated expression of mir-424 in CRC tissues in this study was in consistent with the other two studies based on Chinese patients’ samples[11,40]. However, no other studies reported the altered expression level of mir-424 in CRC. The discrepancy of miRNA expression profile between studies might be attributed to the sample size, genetic background, clinical characteristics or different technique platforms. Strikingly, it was found that mir-7, an up-regulated miRNA with high fold -change in this study (fold-change = 3.60) was not reported as a dysregulated miRNA in CRC in previous studies. In studies with other tumors, an elevated expression level of mir-7 was an indicator of poor disease-free survival in non-small cell lung carcinoma [41], and correlated with a larger size of tumor and poor tumor grade in breast cancer tissues [42] while some reports stated that mir-7 inhibits EGFR and the AKT pathway, and the expression level of mir-7 was down-regulated expression in gilobastoma [43,44]. It seems that the role of mir-7 differs in various types of cancer, suggesting that the expression and function of miRNAs is tissue cell specific. The expression level of mir-7 and its potential targets in CRC are worthwhile for further investigation

In this study, an important note should be added that a very strict criterion was adopted to identify the miRNA targets. Briefly, the target mRNA should be differentially expressed, significantly anti-correlated with its miRNA regulator, and predicted by two databases, TargetScan and miRanda. Based on this strict criterion,our final identified miRNA-mRNA pairs have a high probability of involvement in CRC tumorigenesis. The following analysis will support this high probability. On one hand, comparing the mRNA targets (n = 4338) of the dysregualted miRNAs (n = 32) predicted just by TargetScan, only a small portion of the target genes (244/4338, 5.6 %) were significantly differentially expressed in CRC, and were selected for further analysis, while a great number of mRNAs (4094/4338, 94.4 %) were not differentially expressed in CRC tissue, and were not selected for further analysis. The fact that these 4094 genes were predicted as miRNAs targets but not significantly differentially expressed, suggested that the expression of these 4094 mRNAs might not be regulated by the 32 dysregulated miRNAs, but by other mechanisms such as regulation by other miRNA, methylation, cis-regulatory elements, etc. which may act and counteract the regulation of miRNA. On the other hand, Pearson’s correlation analysis selected 17360 miRNA-mRNA pairs whose expression levels were significantly inversely correlated, however only 72 target relationships of miRNA were finally identified. The existence of the rest of 17288 miRNA-mRNA pairs may be caused by the indirect regulation.

Another interesting finding was that the expression values of mir-29a showed a narrower range of change than those of its predicted target, KLF4. To rationalize this finding, it is hypothesized that the KLF4 mRNA expression was regulated by many other factors including other miRNAs. Besides mir-29a, KLF4 was also identified as a target gene of mir-7, an up-regulated miRNA with high fold-change. The unparalleled change of miRNA and its target mRNA expression levels could be caused by the two miRNA (mir-29a and mir-7) targeting one gene (KLF4). Inspired by this finding, the relationship between fold-change of mRNAs expression and the number of corresponding miRNA regulators were further analyzed. In the identified 72 miRNA-mRNA pairs, three groups can be classified: 1) 2 mRNAs were regulated by 3 miRNAs, 2)10 mRNAs were regulated by 2 miRNAs, and 3) the rest (60 mRNAs) were regulated by 1 miRNA. However, the fold-changes of the gene mRNA expression between these three groups do not reach the statistical significance, indicating the gene expression regulation is very complex and dynamic with impact from multi-factors.

miRNAs regulate diverse biological processes via regulating the expression of target genes. In this study, only the Wnt signaling pathway, which had been proved to be aberrantly activated in CRC [45,46], was found to be affected through pathway analysis of the miRNA targets. Our study revealed that 4 down-regulated mRNA and an up-regulated miRNA regulated by 5 dysregulated miRNA in CRC respectively were involved in the Wnt signaling pathway, and shed light on the miRNA regulation of Wnt signaling pathway.

Nevertheless, the orchestration of these regulatory pairs of miRNA-mRNA differed in different diseases, presenting the disease-specific expression profiles and composing a complex regulatory network. Although many methods had been used to identify the regulatory pairs of miRNA-mRNA in diseases such as microarray and software-prediction, no method possessed the capacity to determine all miRNA-mRNA regulatory pairs. Moreover, since the anti-correlationship of the miRNAs-mRNA expression levels was used as the screening criteria, only those target genes regulated through mRNA degradation could be identified here, target genes regulated by miRNAs through translation inhibition would be lost.

In summary, these new findings of pairs of miRNA-mRNA provide some hints for the mechanisms of CRC tumorigenesis and were worthwhile for further functional analysis. The more complex regulatory networks call for more research in the future.

## **Conclusions**

Based on this research, seventy-two miRNA-mRNA pairs combined by 22 dysregulated miRNAs and their 58 target mRNAs identified by the multi-step approach appear to be involved in CRC tumorigenesis. The identified miRNA-mRNA pairs are worthwhile for further functional studies.

## **Methods**

### **Patients and clinical samples**

Forty CRC tissues and matched adjacent normal colorectal mucosa tissues (at least 5 cm from the cancer) were obtained from patients who underwent surgical excision for CRC at Xinhua Hospital, School of Medicine, Shanghai Jiaotong University. The samples were collected according to the following histological prerequisites: the CRC tissues had more than 80 % tumor cells; the matched adjacent normal colorectal mucosa tissues had normal mucosal structure without dysplastic cells. The samples were snap frozen in liquid nitrogen in 5 minutes after surgical resection and were then stored at −80°C temperature until RNA extraction. Informed consents were obtained from all the patients and the study was approved by hospital ethics committee.

### **RNA extraction**

Total RNA was extracted and isolated using mirVana TM RNA Isolation Kit (Applied Biosystem p/n AM1556) following the manufacturer’s instructions. The concentration of RNA was measured by NanoDrop spectrophotometer. Total RNA concentration ranged form 100 ng/ul to 1ug/ul. The RNA quality was evaluated by Agilent 2100 Bioanalyzer; RNAs with RIN ≥ 7.0 and 28 S/18 S > 0.7 were accepted for microarray analysis.

### **MicroRNA microarray procedure**

8 colorectal cancer tissues and their corresponding adjacent normal tissues ere selected for microRNA microarray analysis from the 40 matched tissues pairs of CRC tissues and their adjacent normal colorectal mucosa tissues. These 8 pairs of samples shared similar clinical and pathological features that they were all the moderately differentiated, non-mucinous adeno-carcinoma and micro-satellite stable. The total RNA was analyzed by microarray (Agilent V12.0) according to the manufacture’s protocol. In brief, 100 ng of total RNA was labeled with Cy3, and then hybridized on a miRNA array (8*15 K,Agilent V12.0) using Agilent’s miRNA Complete Labeling and Hyb Kit (p/n 5190–0456). Microarray after hybridization was washed by GE Wash Buffer 1 and GE Wash Buffer 2 (Gene Expression Wash Buffer kit, Agilent p/n 5188–5327). The slides were then scanned in microarray scanner by Agilent scan control software Version A7.0. The data collection, background subtraction and with-in array normalization were carried out by Agilent Feature Extraction (FE) software version 9.5.3. After Between-array normalization and signal filtration (IsGeneDetected, WellAboveNeg) performed by RMA algorithm [28], the data was analyzed by T-test and Significant Analysis of Microarray analyses(SAM) [29] to identify differentially expressed miRNAs. miRNAs with false discovery rate (FDR) < 0.05 using student’s t-test and q-value < 0.05 for SAM were identified as differentially expressed genes.

### **mRNA microarray procedure**

An amount of 2 ug of total RNA isolated from CRC tissues and corresponding normal tissues were reverse-transcribed into cDNA, and labeled with Cy3 or Cy5. The labeled cDNA was then hybridized on a Whole Human Genome Agilent 4X44K Oligonucleotide Microarray (Agilent) according to the manufacture’s protocol using miRNA Complete Labeling and Hyb Kit. The chips were washed using Agilent Gene Expression Wash Buffer Kit (Agilent 5188–5327). Scanning of the microarrays was carried out immediately using the Agilent G2565BA Microarray Scanner System. Images were auto gridded, analyzed and data extracted using Agilent Feature Extraction software (version 9.5.3). Raw expression data generated by the Feature Extraction software was imported into R using the LIMMA package in Bioconductor [30]. The intensity distributions within and between arrays were normalized using the quantile scaling algorithm in LIMMA, and the log-2 based data was then filtered on flags (IsFound, IsWellAboveBG, IsSaturated) to identify differentially expressed features between colorectal cancer sample and normal colon mucosa. T-test and Significant Analysis of Microarray analyses (SAM) [29] were carried out to identify dysregulated mRNAs.

Similar to the criteria of identifying dysregulated miRNAs, mRNAs with FDR <0.05 for student’s t-test and q-value < 0.05 for SAM were identified as differentially expressed genes.

### **Target predicting**

TargetScanHuman v. 5.1 [20] (http://www.targetscan.org/; Whitehead institute for Biomedical Research) and Mirnada [23] were used to predict the target genes of miRNAs

### **Pathway enrichment analysis**

The pathway enrichment analysis was performed using online database. The Database for Annotation, Visualization and Integrated Discovery (DAVID) v 6.7 [35].

### **qRT-PCR**

Both mRNA and miRNA expression level were detected in 40 CRC tissues and their corresponding adjacent normal tissues by qRT-PCR method using the ABI Detection Kit. For mRNA expression detection, 2 ug of total RNA was reverse transcribed by MMLV transcription according to manufacture’s protocol. The cDNA was analyzed by TaqMan assays. qRT-PCR was performed by ONE-STEP PLUS system (ABI). The primers and probes of qRT-PCR were listed in Additional file 1: Table S3. For miRNAs expression detection, a 15ul of reverse transcription mixture containing 3ul of RT primer, 5 ng of total RNA and 50U of MultiScribe Reverse Transcriptase, 1.5ul Reverse Transcription Buffer (10×), 0.15ul 1 dNTPs (00 mM), 4U of RNase Inhibitor was incubated in PCR tube for 16°C 30 min, 42°C 30 min, 85°C 5 min. The cDNA product were then stored at −4°C. The real-time PCR was performed by ONE-STEP PLUS system (ABI). A total volume of 20 ul reaction mixture included 1.0ul TaqMan Small RNA Assay (20×) ,1.33 ul of Product from RT reaction, 10.0 ul TaqMan Universal PCR Master Mix II (2×), 7.67 ul Nuclease-free water. The reactions were incubated in 96-well optical plate for 95°C 10 min; 95°C 15 s, 60°C 60s with 40 cycles.

The expression level of each mRNA and miRNA was calculated by threshold cycle (CT). The relative expression level was calculated using –ΔΔCT. A median expression sample among all samples was chosen as a calibrator, and U6 as endogenous control for miRNAs expression, beta-actin for mRNAs expression.

### **Cell culture**

Human colorectal cell line HCT 116 was purchased from the Type Culture Collection of the Chinese Academy of Sciences, Shanghai, China. The cell line was incubated in Dulbecco's modified Eagle's medium (DMEM, Invitrogen, Carlsbad, CA, USA) with 10 % FBS.

#### **Transfection**

The hsa-mir-29a mimics, mimics control, hsa-mir-29a inhibitor (anti–miR-95, chemically modified antisense oligonucleotides designed to target specifically against mature miR-29a), miRNA inhibitor control were designed and synthesized from Ribobio (Guangzhou, China). The 4 × 10^6^ of HCT 116 cell were grown in 6-well plates for 24 hours before transfection. The transfection was performed by Roche X-tremeGENE DNA Transfection Reagents according to manufacturer’s protocol. Every experiment was performed in triplicates in 6-well cell culture plates with the appropriate controls. Cells were harvested 48 h after transfection.

### **Western blot**

Total proteins from cultured cells were extracted by RIPA Lysis Buffer (Beyotine, Shanghai, China). 40 ug of proteins were electrophoresed through 10 % SDS polyacrylamide gels and were transferred to a PVDF membranes (Millipore) The membranes were incubated with anti-KLF4 Rabbit polyclonal antibody (ab26648,1:1000, abcam), anti-GADPH antibody was chosen as an endogenous control. Membranes were probed with the appropriate secondary antibodies linked to horseradish peroxidase. Antibody binding was visualized using ECL.

### **Statistical analysis**

Statistical analysis was performed using SPSS15.0. Values were expressed as mean ± standard deviation for parametric data. Difference between groups was compared using student’s *t*-test. Correlation between miRNA and mRNA expression profiles was calculated using Pearson’s correlation. *P* value less than 0.05 was considered as statistically significant. The statistical analysis of microarray analysis was described elsewhere.

## **Authors’ contributions**

JF, JG and LC designed the study and authored the manuscript. PD, JL and WC collected the samples. GW and WT performed the qRT-PCR analysis. WT carried out transfection assays. PD, JF and JG carried out the microarray test and the bioinformatics analysis. LC, PD, JG and JF analyzed data. All authors read and approved the final manuscript. JF and WT contributed equally to this work.

## Supplementary Material

Additional file 1 Supplemental tables and figures Click here for file

Additional file 2 Differentially expressed mRNA in CRC tissues Click here for file

Additional file 3 Significantly anti-correlated miRNA-mRNA pairs Click here for file

Additional file 4 Target mRNAs of up-regulated miRNAs Click here for file

Additional file 5 Target mRNAs of down-regulated miRNAs Click here for file

Additional file 6 Significantly enriched GO biological processes Click here for file
