# Supplementary material for: Identifying MicroRNA-mRNA regulatory network in colorectal cancer by a combination of expression profile and bioinformatics analysis
Source: BMC Syst Biol. 2012 Jun 15;6:68. doi: 10.1186/1752-0509-6-68 (PMC3418553; doi:10.1186/1752-0509-6-68)
Supplement: Additional file 1 — Supplemental tables and figures [file 1752-0509-6-68-S1.pdf]

**Table S1 Clinical features of 8 CRC cases for microarray test**

| Case | Age | Gender | Location [a] | Differentiation | Pathological features | T | N | M | Stage [b] |
|---|---|---|---|---|---|---|---|---|---|
| 577 | 60 | Male | R | moderate | Non-mucinous, Tubular | 3 | 0 | 0 | II |
| 581 | 64 | Male | R | moderate | Non-mucinous, Tubular | 4 | 2 | 0 | III |
| 590 | 65 | Female | S | moderate | Non-mucinous, Papillary & Tubular | 4 | 2 | 0 | III |
| 601 | 88 | Female | R | moderate | Non-mucinous, Tubular | 2 | 0 | 0 | I |
| 661 | 78 | Female | T | moderate | Non-mucinous, Tubular | 4 | 0 | 0 | II |
| 698 | 74 | Male | S | moderate | Non-mucinous, Papillary & Tubular | 3 | 0 | 0 | II |
| 702 | 76 | Female | R | moderate | Non-mucinous, Tubular | 4 | 0 | 0 | II |
| 705 | 74 | Female | R | moderate | Non-mucinous, Tubular | 3 | 1 | 0 | III |

a, R, rectum. S, Sigmoid colon. T, Transverse colon
b, the JACC classification

**Table S2 Differentially expressed miRNAs in CRC compared with adjacent normal tissues detected by microarray test**

| microRNA | Fold change（CRC *VS.* normal） | up/down regulated | FDR（paired *t* test) | q-value (%)（paired SAM） |
|---|---|---|---|---|
| miR-424 | 4.596 | Up | 0.004 | 0.000 |
| miR-224 | 4.572 | Up | 0.000 | 0.000 |
| miR-96 | 4.354 | Up | 0.009 | 0.000 |
| miR-223 | 3.758 | Up | 0.025 | 0.000 |
| miR-7 | 3.360 | Up | 0.009 | 0.000 |
| miR-21 | 3.243 | Up | 0.005 | 0.000 |
| miR-19a | 2.664 | Up | 0.036 | 0.000 |
| miR-17 | 2.591 | Up | 0.008 | 0.000 |
| miR-20a | 2.578 | Up | 0.005 | 0.000 |
| miR-19b | 2.573 | Up | 0.009 | 0.000 |
| miR-106b | 2.164 | Up | 0.009 | 0.000 |
| miR-29b | 1.870 | Up | 0.007 | 0.000 |
| miR-93 | 1.763 | Up | 0.011 | 0.000 |
| miR-27a | 1.614 | Up | 0.035 | 0.000 |
| miR-25 | 1.587 | Up | 0.003 | 0.000 |
| miR-29a | 1.337 | Up | 0.007 | 0.000 |
| let-7g | 0.810 | Down | 0.019 | 1.581 |
| let-7b | 0.734 | Down | 0.022 | 0.858 |
| miR-186 | 0.702 | Down | 0.030 | 0.000 |
| let-7c | 0.700 | Down | 0.035 | 0.858 |
| miR-320b | 0.656 | Down | 0.019 | 0.000 |
| miR-574-3p | 0.623 | Down | 0.009 | 0.000 |
| miR-342-3p | 0.500 | Down | 0.007 | 0.000 |
| miR-10b | 0.425 | Down | 0.006 | 0.000 |
| miR-30a | 0.389 | Down | 0.029 | 0.000 |
| miR-378 | 0.385 | Down | 0.016 | 0.000 |
| miR-150 | 0.327 | Down | 0.010 | 0.000 |
| miR-497 | 0.320 | Down | 0.025 | 0.000 |
| miR-375 | 0.319 | Down | 0.009 | 0.000 |
| miR-195 | 0.299 | Down | 0.018 | 0.000 |
| miR-145 | 0.191 | Down | 0.017 | 0.000 |
| miR-133b | 0.183 | Down | 0.022 | 0.000 |

**Table S3　Clinical data of the 40 cases for qRT-PCR validation test**

| Characteristics | | Number | % |
|---|---|---|---|
| **Age (years)** | | | |
| | < 60 | 14 | 35 |
| | >=60 | 26 | 65 |
| **Sex** | | | |
| | Male | 19 | 47.5 |
| | Female | 21 | 52.5 |
| **Invasion** | | | |
| | T1 | 1 | 2.5 |
| | T2 | 5 | 12.5 |
| | T3 | 15 | 37.5 |
| | T4 | 19 | 47.5 |
| **Lymph node metastasis** | | | |
| | N0 | 23 | 57.5 |
| | N1 | 6 | 15 |
| | N2 | 11 | 27.5 |
| **Metastasis** | | | |
| | M0 | 34 | 85 |
| | M1 | 6 | 15 |
| **Stage**[a] | | | |
| | I | 6 | 15 |
| | II | 15 | 37.5 |
| | III | 13 | 32.5 |
| | IV | 6 | 15% |
| **Location** | | | |
| | Ascending colon | 9 | 22.5 |
| | Transverse colon | 1 | 2.5 |
| | Descending colon | 1 | 2.5 |
| | Sigmoid colon | 9 | 22.5 |
| | Rectum | 20 | 50 |
| **Mucinous carcinoma** | | | |
| | mucinous carcinoma | 6 | 15 |
| | non-mucinous carcinoma | 34 | 85 |
| **Micro-satellite** | | | |
| | MSS | 24 | 60 |
| | MSI-H | 16 | 40 |

a, the JACC classification

**Table S4 Primers and probes used for qRT-PCR**

| Gene | | Sequence of primers and probes |
|---|---|---|
| SFRP1 | FP | ACGTCTGCATCGCCATGA |
| | RP | CTCAGATTTCAACTCGTTGTCACA |
| | Probe | FAM-CAAGCCCCAAGGCACAACGGTGT-BHQ1 |
| KLF4 | FP | CGCTCCATTACCAAGAGCTCAT |
| | RP | CGATCGTCTTCCCCTCTTTG |
| | Probe | FAM-TTCCTGCATGCCAGAGGACCC-BHQ1 |
| RFN138 | FP | TGCTCAGGAGGTGCTCAAAACG |
| | RP | CATTGCAGTCAGGAAACATTTTCT |
| | Probe | FAM-CGTGCGGACCACGGCCTGTC-BHQ1 |
| SFRP2 | FP | TGCTTGAGTGCGACCGTTT |
| | RP | CAAAGCGTTTCCATTATGTCGT |
| | Probe | FAM-ACAACGACCTTTGCATCCCCCTCG-BHQ1 |

**TaqMan probes were labeled by a 5'FAM reporter and 3'BHQ1 quencher.**

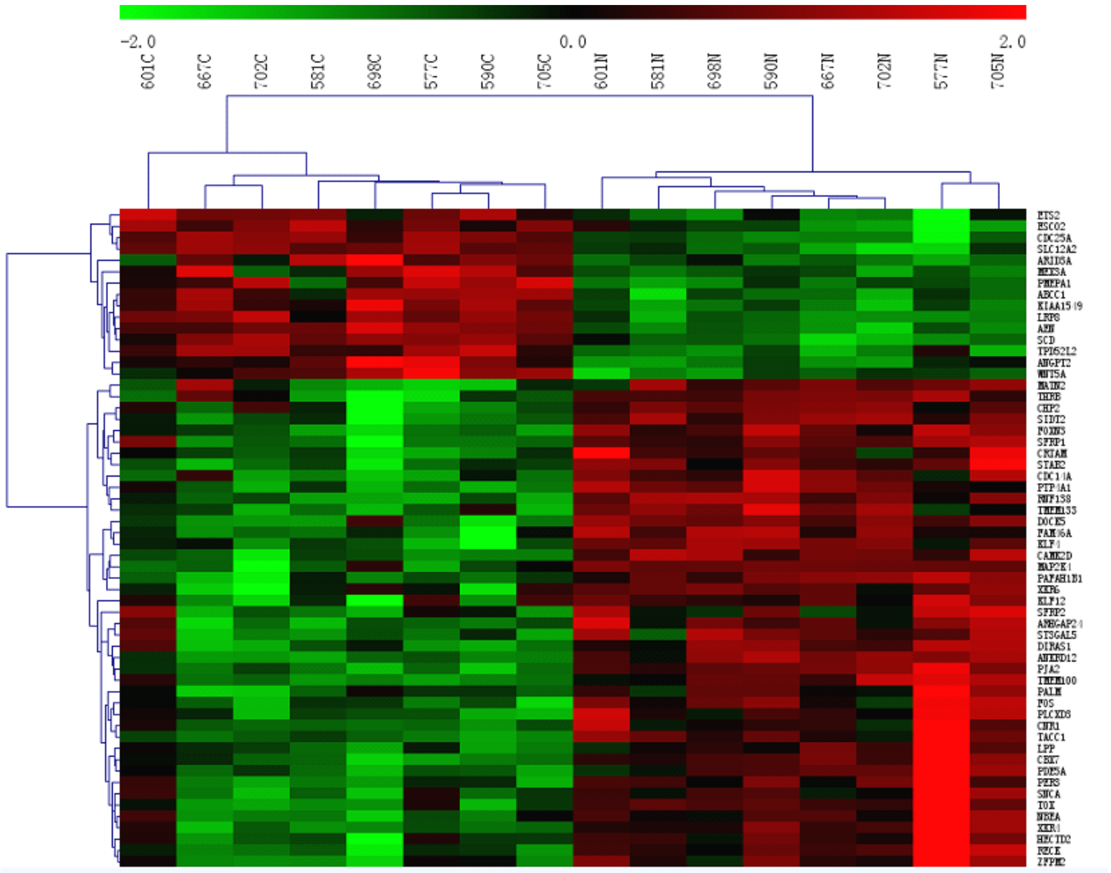


**Figure S1-Heatmap of 58 target genes identified in our study**

FDR<0.05 for students t-test and q-value<0.05 for SAM were considered as significantly differentially expressed. The data were z-score transformed and were indicated by the color bar above the heatmap.
